# Renaissance of armored immune effector cells, CAR-NK cells, brings the higher hope for successful cancer therapy

**DOI:** 10.1186/s13287-021-02251-7

**Published:** 2021-03-22

**Authors:** Faroogh Marofi, Heshu Sulaiman Rahman, Lakshmi Thangavelu, Aleksey Dorofeev, Favian Bayas-Morejón, Naghmeh Shirafkan, Navid Shomali, Max Stanley Chartrand, Mostafa Jarahian, Ghasem Vahedi, Rebar N. Mohammed, Somayeh Shahrokh, Morteza Akbari, Farhad Motavalli Khiavi

**Affiliations:** 1 Department of Hematology, Faculty of Medicine, Tabriz University of Medical Sciences, Tabriz, Iran; 2 Immunology Research Center, Tabriz University of Medical Sciences, Tabriz, Iran; 3 Department of Physiology, College of Medicine, University of Suleimanyah, Sulaymaniyah, Iraq; 4 Associate professor, Department of Pharmacology, Saveetha Dental College and Hospital, Saveetha Institute of Medical and Technical Sciences, Saveetha University, Chennai, India; 5 Department of Propaedeutics of Dental Diseases, I.M. Sechenov First Moscow State Medical University (Sechenov University,), Moscow, Russian Federation; 6 Center for Research and Biotechnological Development, Research Department, Bolivar State University, Faculty of Agricultural Sciences, Natural Resources and the Environment, CP 020150 Guaranda, Ecuador; 7 DigiCare Behavioral Research, Casa Grande, AZ USA; 8 German Cancer Research Center, Toxicology and Chemotherapy Unit (G401), 69120 Heidelberg, Germany; 9 Department of Immunology, Faculty of Medicine, Zanjan University of Medical Sciences, Zanjan, Iran; 10 College of Veterinary Medicine, University of Sulaimani, Suleimanyah, Iraq; 11 Department of Pathobiology, Faculty of Veterinary Medicine, University of Shahrekord, Shahrekord, Iran; 12 Department of Virology, Pasteur Institute of Iran (IPI), Tehran, Iran

**Keywords:** CAR, NK cells, T cells, Immunotherapy

## Abstract

In recent decades, a new method of cellular immunotherapy was introduced based on engineering and empowering the immune effector cells. In this type of immunotherapy, the immune effector cells are equipped with chimeric antigen receptor (CAR) to specifically target cancer cells. In much of the trials and experiments, CAR-modified T cell immunotherapy has achieved very promising therapeutic results in the treatment of some types of cancers and infectious diseases. However, there are also some considerable drawbacks in the clinical application of CAR-T cells although much effort is in progress to rectify the issues. In some conditions, CAR-T cells initiate over-activated and strong immune responses, therefore, causing unexpected side-effects such as systemic cytokine toxicity (i.e., cytokine release syndrome), neurotoxicity, on-target, off-tumor toxicity, and graft-versus-host disease (GvHD). To overcome these limitations in CAR-T cell immunotherapy, NK cells as an alternative source of immune effector cells have been utilized for CAR-engineering. Natural killer cells are key players of the innate immune system that can destroy virus-infected cells, tumor cells, or other aberrant cells with their efficient recognizing capability. Compared to T cells, CAR-transduced NK cells (CAR-NK) have several advantages, such as safety in clinical use, non-MHC-restricted recognition of tumor cells, and renewable and easy cell sources for their preparation. In this review, we will discuss the recent preclinical and clinical studies, different sources of NK cells, transduction methods, possible limitations and challenges, and clinical considerations.

## Introduction

As we expand our knowledge and understanding of how the immune system acts against cancer, we are more likely to believe in the immune system’s power. Therefore, in recent decades, we relied more on novel immune-based therapies than the classic chemical drugs (chemotherapies) to treat cancer. Various novel immunotherapy methods are in development to achieve effective and durable therapies for patients with hardly treatable or refractory cancers [1]. Cancer immunotherapy exploits the body’s immune system to fight against cancer and is classified by three branches: immune checkpoint inhibitors (ICIs), adoptive cell therapies (ACTs), and tumor vaccines [2, 3]. Cancer immunotherapy can help patients with advanced tumors or recurrent cancers [4].

ICIs block the checkpoint receptors/ligands on immune cells and cancer cells then prompt the killing mechanisms in immune effector cells. This type of immunotherapy was found to be advantageous in various malignancies, e.g., Hodgkin’s lymphoma [5, 6]. ICIs have gained much attention over the past years for their great therapeutic potential in various cancers. Several ICIs have been investigated or are being tested. Novel therapeutic antibodies have been developed relying on NK cell-based ICI such as Lirilumab, Monalizumab, Lacutamab, Etigilimab, Tiragulomab, Relatlimab, Eftilagimod alpha, Cobolimab, Samalizumab, Magrolimab, Ipilimumab, Nivolumab, Pembrolizumab, Atezolizumab, Durvalumab, Avelumab, Dostarlimab, Spartalizumab, Tislelizumab, Lodapolimab, Enoblituzumab, Orlotamab, and many other developed/under development therapies [7–10]. The inhibitory receptors on NK cells with promising potential to develop ICIs are classical NK cell receptors KIRs, LIRs, and NKG2A. There are also some other important ICIs such as CTLA-4, PD-1, and recently determined B7-H3, and LAG-3, TIGIT and CD96, TIM-3, and recently characterized checkpoint-members of the Siglecs family (Siglec-7/9), CD200 and CD47. Blocking these ICIs on NK cells push the balance of activation-inhibition of NK cells toward activation due to lack of inhibitory signals. Activated NK cells have substantially more cytolytic/killing power and therefore can detect and attack cancer cells more efficiently. The combination of blocking ICIs on NK cells with CARs can lead to a very efficient and cancer-redirected cytolytic activity of CAR-NK cells [11, 12].

In addition, Bi- and tri-specific antibodies as the novel immunotherapy strategy can engage the immune effector cells toward tumor cells by binding simultaneously to both cells. Then, the forced proximity between effector cells and tumor cells causes the release of cytotoxic molecules and apoptosis of tumor cells. This method showed good anticancer activity against several types of non-Hodgkin lymphomas (NHL) such as diffused large B cell lymphoma (DLBCL) and follicular lymphoma (FL) [13–15].

Also, another branch of immunotherapy is comprised of genetic modification of immune cells as living drugs. The method is currently under investigation for understanding its usefulness in the treatment of different cancers particularly hematological malignancies. The first successful forms of transduced cells with CAR were autologous T cells capable of targeting CD19-specific antigen expressed on B cell malignant tumor cells [16]. CAR is an engineered receptor protein capable of specific targeting the antigens introduced to the T cell. Its structure consists of an extracellular antigen recognition domain attached to an intracellular peptide/protein which acts as the intracellular signaling domains [17].

CARs have been successfully utilized in many research and clinical trials to redirect autologous T cells against lymphoid leukemia and lymphoma [18]. CAR-expressed T cells directly recognize the CAR-targeted antigen on cancer cells, and then, T cell activation, the release of cytotoxic molecules, proliferation, cytokine secretion, and immune cells recruitment are triggered spontaneously following the CAR-antigen contact. These types of modified T cells have demonstrated success in the treatment of hematological cancers, including lymphoma, chronic lymphocytic leukemia, and acute lymphoblastic leukemia (ALL) [17].

Therefore, the clinical application of CAR T cells has been mainly constrained to CD19-expressing B cell blood cancers [19–24]. Many research has been conducted to explore CAR-T cells’ suitability to be utilized in other hematological cancers such as Hodgkin and non-Hodgkin lymphoma, multiple myeloma (MM), and acute myeloid leukemia (AML) [25–27]. Although the efficacy of CAR T cell therapy has been good in some types of hematological malignancies, it is important to bear in mind that, with over 100 types of cancer, hematological cancers make only a small part of the diagnosed cases with a cancer and are responsible for only 6% of all reported cancer deaths [28].

CD19-CAR T cell adoptive immunotherapy has achieved successful results in clinical trials in the treatment of ALL and diffuse large B cell lymphoma [29], but there are also substantial disadvantages causing significant side effects such as cytokine release syndrome (CRS), immune hyperactivation, and neurological toxicities [30]. Furthermore, the method has some considerable limitations including the challenges to generate sufficient autologous T cells for each patient, time-consuming and sophisticated preparation procedures, and the high preparation costs which altogether make the method not very cost-effective and easily applicable for health care systems [31]. Considering these limitations, NK cell-based immunotherapy has recently come to light as a potential alternative approach because of its strong antitumor efficiency, no need for prior sensitization, easy preparation process, and better safety profiles in terms of CRS and GvHD. Albeit, allogeneic NK cells, in contrast to primary autologous NK cells and NK cell lines still have the risk of triggering GvHD [32–34].

NK cells are a key member of the innate immune system, and while they have some similarities to cytotoxic T lymphocytes (CTLs) in term of killing features, they have an extra intrinsic capability to detect and kill transformed/mutant cells independent of specific antigen recognition processes indicating MHC-unrestricted cytotoxicity done with the help of a wide range of receptors such as the natural cytotoxicity receptors (NCRs) [24, 35]. Introducing CAR on the surface of NK cells have shown the potential of CAR-NK cells to be used as off-the-shelf cellular immunotherapy which can be administered instantly as clinically required and could resolve some of the difficulties related to logistics and cost s[31]. Expression of CARs in NK cells may grant these cells to more effectively destroy solid tumors that are often more resistant to effector cytotoxic cells in comparison to hematologic cancers (e.g., acute myelogenous leukemia) that are generally more susceptible to NK cell’s cytotoxic fucntions. However, the cytotoxic function of CAR-NK cells is not CAR-restricted; in fact, the CAR plays a recognition-enhancer role for NK cells [36, 37].

In this article, we tend to discuss in the field of recent advances in CAR-engineered NK cell immunotherapy, the advantages of using CAR-NK cells toward CAR-T cells, and different sources of NK cells used for CAR manipulations. Also, various transduction methods to introduce CARs into both primary NK cells and NK cell lines regarding increase efficacy have been discussed. Besides, the results of pre-clinical and clinical studies and the strategies for overcoming challenges and improving safety have been summarized.

### NK cells: history, subsets, and mechanisms of their function

Natural killer (NK) cells were first identified in 1975 as a subpopulation of cells that were different from T and B lymphocytes [38, 39]. For a long time, the functions and biology of NK cells were in the shadow of other immune cells [40]. However, great advances in knowing the NK cells’ biology have revealed important outlooks of their inherent capacity to kill infected or malignant cells without prior sensitization and human leukocyte antigens (HLA)-restriction [38, 41, 42].

NK cells are innate immune cells that reside in the blood, spleen, liver, lung, bone marrow, and to a lesser extent in lymph nodes [43]. They play crucial roles in the immune hemostasis and immune surveillance of tumor cells [44, 45]. Human NK cells are typically characterized by a lack of CD3/TCR molecules while expressing the CD56; thus, they are known for a usual CD56^+^CD16^+^ CD3^−^ phenotype. By considering the expression levels of CD56, NK cells are subdivided into two separate subsets; CD56^bright^ and CD56^dim^ [46]. CD56^dim^ NK cells are almost 90–95% of NK cells in the peripheral blood (PB) and inflammatory sites and are capable of mediating high cytotoxicity responses [47]. Since CD56^dim^ cells also express Fcγ receptor CD16, they can detect and destroy tumor cells or virus-infected cells covered by antibodies and therefore can initiate antibody-dependent cell-mediated cytotoxicity (ADCC) [48]. In contrast, CD56^bright^ NK cells are immature and comprise only about 5–15% of NK cells in PB [49]. CD56^bright^ NK cells are present in secondary lymphoid tissues and are predominantly powerful in cytokine secretion but with limited cytotoxic properties. CD56^bright^ cells have immunoregulatory functions by producing cytokines or chemokines [50, 51] such as IL-12, IL-18, and IL-15 [52]. Given the inherent cytotoxic ability of NK cells, they can release abundant cytotoxic molecules such as perforins and granzymes toward target cells. On the other hand, activated NK cells interact with other immune cells such as dendritic cells (DCs), T cells, and B cells and impress them by secreting various cytokines and chemokines. They can mediate spontaneous cytotoxicity toward abnormal cells and rapidly secrete immune-regulating cytokines such as IFN-γ, TNF-α, and GM-CSF. The cytokines and chemokines released by NK cells help in triggering and adjustment of immune responses against pathogens and malignancies [53, 54].

Unlike T and B lymphocytes, NK cells do not express antigen-specific recognizing receptors (e.g., TCR by T cells). Abnormal cell recognition by NK cells mainly relies on a balance between inhibitory and activating signals. NK cells have a complex collection of receptors capable of signaling either inhibiting or activating messages. The receptor repository of NK cells contains mainly the non-specific primary receptors that make NK cells capable of exerting more general and not antigen- and TCR-restricted recognition (unlike the T cells). The receptors can be considered as a kind of pattern recognition receptors, not antigen-specific receptors [55]. In other words, NK cells at the same time express both inhibitory and activating receptors, and their cytotoxic functions can be initiated when a balance between the signaling network of inhibitory receptors and activating immune receptors is interrupted [56]. Inhibitory receptors have a key role in the selectivity function of NK cells by developing NK cells tolerance toward healthy normal cells. When those germline-encoded inhibitory receptors bind to major histocompatibility complex (MHC)-I on a normal cell, a cascade of intracellular signaling mechanisms is triggered: SH-2 containing protein tyrosine phosphatase (SHP)-1 and SHP-2 are recruited to immunoreceptor tyrosine-based inhibitory motifs (ITIMs) in their cytoplasmic parts. These events oppose the kinases activated by activating receptors; thus, at the end, NK cells remain inactive [57]. Nevertheless, when abnormal conditions such as infectious disease or cancer occur, NK cells can be activated due to a lack of MHC-I on abnormal cells and receiving more activating signals [58].

The inhibitory receptors interacting with MHC molecules contain a large repertoire of receptors. The repertoire includes Ig-like receptor (KIR) on human NK cells while Ly49, a family of polymorphic type II proteins with C-type lectin domains, on murine NK cells [47, 59] and the heterodimeric C-type Lectin receptor (CD94/NKG2A) which is similar in both human and mouse species [58].

Members of the KIR family normally recognize a subset of classic MHC-I molecules, including human leukocyte antigen (HLA-A, -B, and -C) [44]. In a similar manner, Ly49 recognizes a subset of H-2 molecules [60]. However, CD94/NKG2A can link to non-classical MHC-I molecules such as Qa-1b in mice and HLA-E in humans [61]. On the other hand, the activating receptors consist the natural cytotoxicity receptors (NCRs) NKp46, NKp30, NKp44, and the C-type lectin-like activating immunoreceptor NKG2D and 2B4 (CD244), Fcγ RIIIA (CD16), and some others [62, 63].

Based on mentioned points above, NK cells recognize target cells through one of these models: “missing-self recognition,” “stress induced-self recognition,” and “non-self-recognition.” Since MHC molecules expression on host cells downregulated or cell transformation occurred, NK cells activated in a process called “missing-self recognition” [64]. Under conditions that NK activating receptors distinguish upregulated self-proteins in transformed or infected cells, “Stress induced-self recognition” is a way of NK cells activation [64]. And finally, “Non-self-recognition” refers to the status that foreign pathogen-encoded molecules are identified by some NK activating receptors [65].

### CAR-NK cell immunotherapy

It is more than a decade that NK cells have been reported as efficient effector cells in adoptive immunotherapy [66, 67]. Unlike T cell-mediated immunotherapy, anti-tumor responses of these cells are usually effectual due to distinct characteristics of NK cells including antigen-independent recognizing and no induction of immune-mediated dysfunctions such as GvHD [68, 69].

In recent years, genetic manipulation of NK cells has been considered to improve cytotoxicity, tumor-targeting capability, and persistence in the tumor microenvironment (TME) [70]. One of the examples of these modifications is CAR transduction in NK cells [71]. The introduction of CAR-NK cells as a novel method of cellular immunotherapy has provided us new insights and opportunities to fight against malignancies and infectious diseases [72–74].

**Fig. 1:**
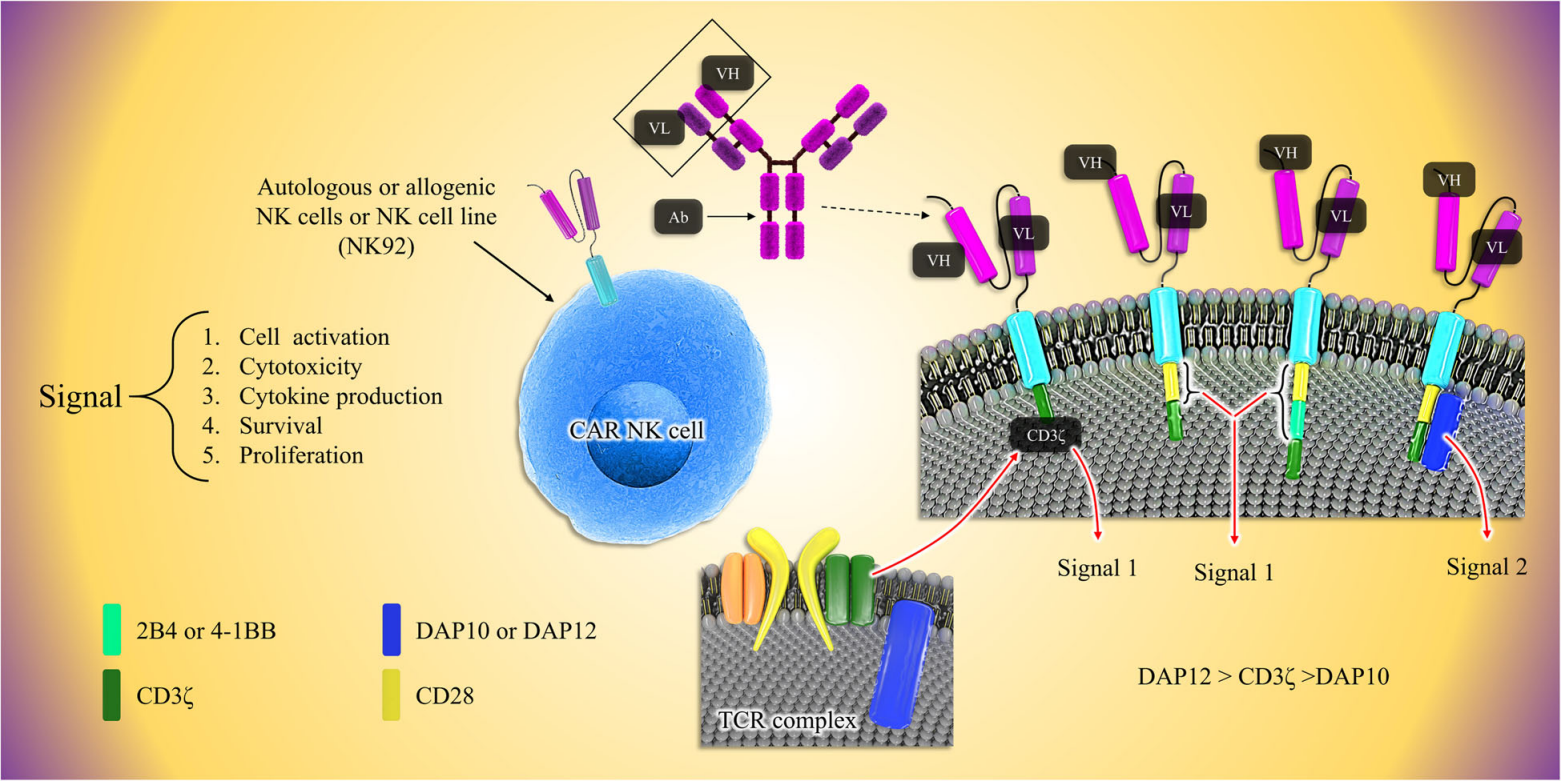
The fundamentals of CAR engineering of three generations of CAR-NK cells. CAR molecules on NK cells consist of three main parts: an antigen detection domain (e.g., ScFv), a transmembrane domain, and the signaling domain. First-generation CARs consist of CD3ζ as the signaling domain. Second-generation CARs have the CD-28 or a combination of CD-28 and a second additional signaling molecule: 4-1BB. As an example of 3rd generation, a CAR comprising an activating receptor; NKG2D can be constructed containing the signaling molecules: CD3ζ and DAP10/12. It was found that CD3ζ has better signaling properties than DAP10 and also it seems that DAP12 may activate NK cells better than both CD3ζ and DAP10. NK cells antigen binding or receiving activation signal by a bunch of activator receptors such as NKG2D leads to signal transduction, NK cell activation, the release of cytolytic enzymes, cytokine production, and also NK cells expansion and maintenance

Similar to CAR-T cells, CAR-engineered NK cells have three parts; an extracellular region comprised of a single-chain variable fragment (scFv) to target a tumor-specific antigen, transmembrane, and intracellular domains [45]. First-generation CAR-NK cells only have a CD3ζ chain as the signaling domain [75, 76]. More investigations have shown that adding one or two co-stimulatory domains improves the potency and cytotoxicity of CAR-NK cells. Later in second- and third-generation, besides the primary CD3ζ chain, NK cells were also equipped with additional specific signaling endodomains such as CD28, 2B4, 4-1BB, OX-40 DAP10, and DAP-12 [77–80] (Fig. 1). Compared to CAR-T cells, there are many advantages in applying CAR-transduced NK cells in targeted immunotherapy. First, because of no requirement of NK cells to HLA matching, they are tolerated well and do not lead to GVHD [81, 82]. Second, CAR-NK cells do not cause severe toxic effects; activated NK cells produce useful and safe cytokines such as IFN-γ and GM-CSF while CAR-T cells principally release pro-inflammatory cytokines, such as TNF-α, IL-1, and IL-6 which then resulted in CRS [83–85]. Third, there are various sources of NK cells such as PB, umbilical cord blood, human embryonic stem cells (hESCs), induced pluripotent stem cells (iPSCs), and even immortal cell lines [64, 86]. Finally, because of the short life of NK cells, on-target/off-tumor effects occur rarely [87]. Hence, these advantages have led to optimism and enthusiasm for using CAR-NK cells for immunotherapy instead of utilizing CAR-T cells.

### Sources for preparing CAR-expressing NK cells

There are different sources that functional NK cells can be extracted and generated from [18]. One of these sources is PB-NK cells, which can be isolated from peripheral blood samples taken from donors or on a larger scale by apheresis. The advantages of these cells are their high expression of activating receptors, such as CD16, NKp44, and NKp46 and KIRs [88] permitting them to be activated readily, and additionally, PB-NK cells can be easily expanded in vivo without further stimulation processes [89].

However, working with blood NK-cells is challenging because of some difficulties in the collection, in vitro expansion, and transduction of these cells [24]. Autologous NK cells can lose their efficacy within the body. The reason behind this issue is that they undergo inactivation processes when they come across to self-MHC presented by cells. On the other hand, using allogenic NK cells have the risk of GvHD even after the CD3 lymphocytes have been depleted [90].

The second source of NK cells is derived from immortal NK cell clones. These cell lines have good cytotoxic potential. Some of these cell lines are NKG, NKL, KHYG-1, HANK-1, YT, NK-YS, YTS, SNK-6, IMC-1, and NK-92 cells [24]. They are more suitable sources for CAR-engineering techniques than PB-NK cells due to their homogeneous cell population, easy expansion procedures, and no need for donors [91]. NK-92 cell line was first isolated from a male patient with non-Hodgkin’s lymphoma [92] and was the most frequently used cell line for CAR-NK cell preparation in studies and even has been utilized in clinical trials (NCT00900809 and NCT00990717).

However, the strengths and weaknesses usually come together, and the NK cell lines suffer from some disadvantages such as the lack of CD16 expression in most cell lines and non-permanent expression or lack of important activating receptors like natural cytotoxicity receptors (NCRs) [92, 93]. Furthermore, they might cause multiple cytogenetic abnormalities and suffer from low persistence in vivo [94].

Although in experimental studies NK-92 cells succeed in restraining tumor burden, due to the short lifespan, they lack sufficient long-term therapeutic efficacy in xenograft mouse models [33, 95]. Furthermore, there are some concerns about the tumorigenesis potential of NK-92 cells. To deal with this issue, NK-92 cells are irradiated before injection to patients [96, 97]. Another concern refers to the triggering stroke due to injection procedure and NK-92 cell aggregation in mice [24, 73, 98].

NK-92MI and NK-92CI cells were derived from NK-92 cells and have biological characteristics similar to their progenitors, but are not dependent on IL-2 stimulation [99]. In recent years, NK cells originated from hESCs and iPSCs have been gaining more interest as the sources for producing engineered NK cells. iPSC/hESC-derived NK cells express general NK cell markers such as KIRs, CD16, NKp44, NKp46, NKG2D, and TRAIL, and also, they have shown cytotoxicity against several types of hematological and solid tumors in vitro [100, 101]. Therefore, iPSC/hESC-derived NK cells may be good candidates for CAR engineering to fight against different cancers. In an ovarian cancer xenograft model, CAR-expressing iPSC-NK cells exhibited good antitumor activity, significantly inhibited growth and prolonged survival compared to PB-NK cells, unmodified iPSC-NK cells, or iPSC-NK cells expressing conventional T-CAR (constructed with CD28-4-1BBζ, not the NK cell-derived activating domains) [102]. Interestingly, the in vivo persistent NK cells were significantly increased in the circulation, spleen, and peritoneal fluid in a separate group of tumor-bearing mice post-injection compared to mice injected with PB-NK cells and iPSC-NK cells after 10 days [102]. Also, NK-CAR-iPSC cells targeting CD19 showed remarkable efficiency in controlling CD19+ leukemia progression in humanized mouse models, while less occurrence of side-effects such as CRS [103].

Furthermore, stem cell progenitors have been introduced as a unique source to be differentiated into NK cells. Additionally, NK cells can be directly isolated from the umbilical cord blood (CB). The low immunogenicity of cord blood cells significantly reduces the risk of relapse and GvHD [104]. Recently, CB-NK cells transduced with CAR-CD19, IL-15, and inducible caspase-9 (iC9) suicide gene have shown enhanced persistence and antitumor activity in a Raji lymphoma mouse model [55]. However, further research is needed to determine whether these cells can be properly activated and can elicit efficient cytotoxicity against tumor cells.

### In vitro culturing conditions and expansion of NK cells

Naturally, NK cells have a short lifespan. Primary NK cells or CAR-NK cells can be induced to proliferate and last longer within the body. Some endogenous or exogenous stimulating mediators (e.g., cytokines) can be used to provide stimulus. Using growth/proliferation promoters in vitro requires time-consuming and sometimes high-cost techniques and procedures [105, 106]. Also, isolation and purification of NK cells from sources such as peripheral blood mononuclear cells (PBMCs) and umbilical cord blood is a quite complex process. It needs sophisticated equipment for cell sorting: flow-cytometer. To use a flow-cytometer, further procedures such as sample preparation and multiple antibodies to detect NK cell markers (i.e., CD56, CD16) should be performed [107]. It seems that we need to develop cost-effective methods for in vitro preparation of NK cells and keeping them persistent within the body. Natural/primary NK cells need nutrition-rich mediums such as the stem cell growth medium (SCGM) and as well as the feeder cells such as K562 to accompany, feed, and stimulate them in vitro cell culture. Co-culturing NK cells with irradiated stem cells as feeder cells can also lead to activation of NK cells. Stem cells themselves can be plated in suitable conditions (i.e., stimulatory cytokines and feeder layer) to be differentiated into NK cells. Stimulatory molecules such as the phytohemagglutinin (PHA) and anti-CD3 monoclonal antibody (OKT3) can be used to trigger the proliferation of NK cells [108, 109]. PHA often is used for the activation of human PBMCs. Also, PHA is a good activator of PBMC-derived NK cells in vitro. There are three cytokines with clear boosting activity on NK cells’ function and expansion in vitro: IL-2, IL-3, IL-15, IL-21, and FLT-3L. Each of these cytokines alone or their combination(s) can activate NK cells to proliferate and attack the cancer cells. Inducing endogenous IL-2, IL-15, and IL-21 or administering them exogenously in vivo experiments can lead to NK cell expansion and persistence. IL-2, zoledronate, and Group A streptococcus are potentiating and activating factors for the immune system and immune cells which can also be used to stimulate CB-derived NK cell proliferation/expansion. Unlike the difficulties with PBMC/CB-derived CAR NK cells, we face far fewer problems with CAR-NK cells prepared from cell lines such as NK92 and NK101. The cell lines have good features such as good proliferation rate, easy in vitro expansion and preparation procedures and more durability within circulation [110].

### Transduction methods for CAR-NK cell engineering

For more than a decade, scientists have followed different methods to genetically modify NK cells to utilize them as immunotherapeutic agents in cancer. There are various modification strategies to improve effector functions, persistence, safety, and efficacy of NK cells.

Increased persistence was achieved by genetically manipulating the cytokine-expressing NK cells which as a result, enhanced their stability within TME via self-expression of IL2 and IL-15. Therapeutic efficacy was achieved by the expression of CAR on NK cells to target specific tumor antigens. Better safety can also be obtained by the incorporation of suicide genes into NK cells [111]. In summary, incorporation of a foreign gene into an immune cell requires specific vector systems including (a) viral vectors or (b) non-viral vectors. Despite the difficulties of viral vector transduction in NK cells, the efficacy of these vectors is relatively higher than non-viral systems. It is also interesting to mention that the transduction efficacy in NK cells usually is higher than that in T cells. The most commonly used vectors for introducing CAR genes are viral vectors derived from different virus families such as the retrovirus family; α-, β-, γ-, δ-, and ε-retroviruses [112], spumaviruses, and lentiviruses [113].

One of the advantages of lentiviral vectors over retroviral vectors is their capability to infect both dividing and non-dividing cells. Retroviral vectors can only infect dividing cells [114]. Another benefit is the capability of the lentiviral vector to carry larger transgenes in comparison to retroviral vectors [115]. One issue during the transduction of an NK cell is that the gene-encoding vector can be arbitrarily and stably integrated into the cell’s genetic material. This issue can lead to mutagenesis which was reported in several experiments. In these studies, γ-retroviral vectors were used to find a treatment solution for different diseases such as X-linked severe combined immunodeficiency (X-SCID) and Wiskott-Aldrich syndrome [116–118].

The efficacy of retroviral transduction is very high in expanded primary NK cells with a median of 69% (43–93%) [79], but this gene delivery system has been known for some pitfalls such as insertional mutagenesis, and constant and uncontrolled opposing effects because of improper transgene expression. On the other hand, although lentiviral vectors have a lower risk of inducing insertional mutations, their transduction efficacy in PB NK cells is low; between 8% and 16%. However, the transduction efficacy of these vectors to deliver CAR genes into cord blood-derived NK cells was reported 73% [119]. Due to the drawbacks and safety concerns of viral transduction, a non-viral alternative such as the transposon/transposase system [120, 121] and mRNA transfection [110, 112] have been progressed and tested from the last two decades till now [75, 119]. The non-viral-based gene delivery methods comprise all synthetic gene carriers capable of inducing relatively stable gene expression in target immune cells such as NK cells. The success of non-viral gene delivery is tightly associated with proper selection of two factors: (a) physical delivery methods such as the electroporation and sonoporation, microinjection, and magnetofection and (b) the gene carriers and enhancing buffers such as the biphasic polymers or liposomes or other carriers. These carriers can merge with cell membranes and insert the gene codes into cells. They induce temporary expression of target genes, last a few days, and disappear in dividing cells after a short time [122, 123]. Also, these vectors are inexpensive and have low immunogenicity, but they cannot integrate transgenes into the genome, and they just induce a temporary expression of desired genes [17].

There are also much safer non-viral methods to deliver genetic material into the target cells such as electroporation, sonoporation, microinjection, magnetofection, and patented nucleofection technique. Electroporation-based methods are safer compared to viral methods due to the transient nature of delivered foreign genetic materials (e.g., mRNA), applicable in both dividing and non-dividing cells, and no need for direct manipulation of the cell’s genome [124–126]. This method is based on creating a temporary pore on the cell membrane that provides a transient insertion channel into the cell. However, one of the problems of this method is the possibility of cell damage due to permanent electroporation [127]. As reported recently, mRNA encoding CARs were successfully electroporated into peripheral blood-derived NK cells with an efficiency of up to 81% [128]. The NK cells electroporated with these mRNAs exhibited a distinct increase in their cytotoxicity against B cell malignancies in vitro and in preclinical animal models, which later led to a trial exploring their use in humans [128, 129]. Another noticeable point is that whereas viral-based methods usually need to be performed on dividing cells at their exponential growth phase, the electroporation-based methods do not require the cells to be on their division phase and are successful in both dividing and non-dividing cells. Early studies utilizing the electroporation method for delivery of genetic material into NK cells have shown over 50% of efficacy. This method has been also used for engineering CAR-NK cell targeting CD20 antigen on cancerous cells in B cell non-Hodgkin lymphoma [130] and Burkitt lymphoma [78]. Also, NK cells nucleofected with anti-ROR1 CAR mRNA were used for the treatment of metastatic solid tumors [131, 132].

In the last two decades, an interesting biologic phenomenon was discovered referring to the transfer of intact membrane patches during immune-cell contacts through an immunological synapse (IS) which was later named trogocytosis. It was also found that this mechanism of exchanging cellular components (e.g., the surface receptors) [133] can also be utilized for delivering directly the entire anti-CD19 CARs (not just mRNAs) to NK cells. In a recent study, anti-CD19 CAR-expressing K562 cells were used as donor cells in a co-culture with peripheral blood-derived CD56 + CD3−NK cells. After co-culture, the presence of CAR molecules on NK-cells proved the successful transfer of anti-CD19 CAR from K562 cells to NK-cells through trogocytosis. However, this method is very new and immature and further research is required to prove its clinical relevance [134].

**Fig. 2:**
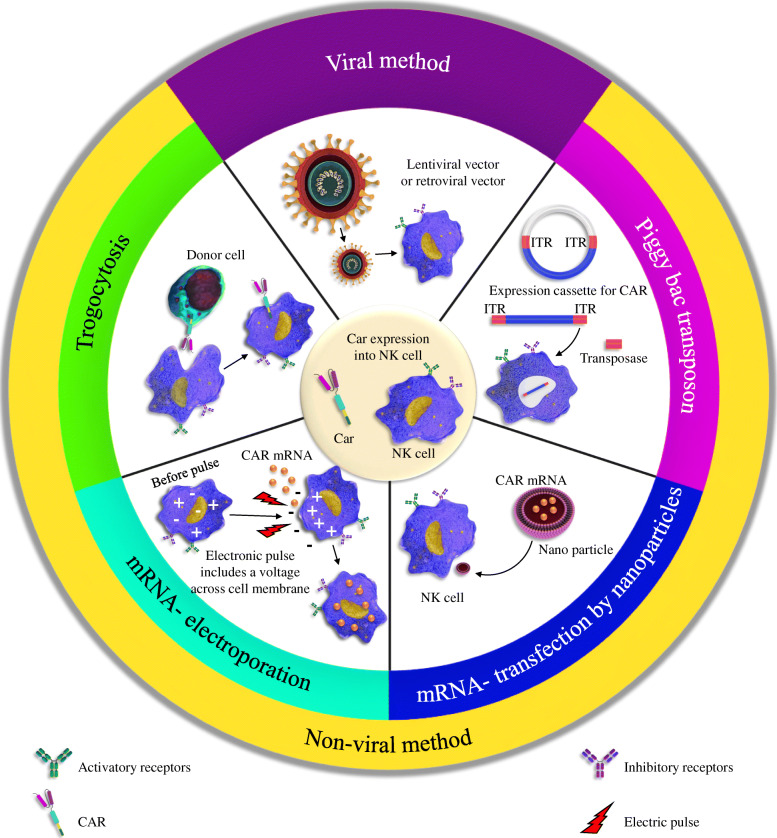
Different approaches for delivery of CAR-containing genes/entire CARs into NK cells. There are two main categories of methods, viral and non-viral. In the viral method, the lentiviral or retroviral vectors carrying CAR genes are delivered into the NK cells. Non-viral methods include various approaches including trogocytosis, the piggyBac transposon, mRNA-electroporation, mRNA transfection, and nanoparticles. The goal of all methods is to equip primary NK cells with CARs

PiggyBac transposon system is another effective non-viral method for genetic element delivery. The system is based on an enzyme (i.e., transposase) that can target-specific sequences on DNA. The enzyme called transposase recognizes the transposon-specific inverted terminal repeat sequences (ITRs) located on both ends of a transposon vector and then simply cut the sequence and paste it somewhere on the target DNA. Therefore, upon recognition, the transposase easily integrates genetic elements from the original sites into chromosomal sites. This creates stable/permanent genomic integration of genomic material [135]. This technique has been also used for CAR modification of T cells [55, 136]. But in terms of NK cells, only two studies are reporting the introduction of CARs to NK cells with the help of the piggyBac transposon system. In one study, Wang et al. engineered NK cells with NKG2D-CAR-presenting vectors with the help of the piggyBac transposon system. They also incorporated DAP10 and CD3ζ as the co-signaling domains within the CAR construct to be used for the transposition process. Besides, they blocked NK-cell-suppressing CD73 markers on cancer cells using the anti-CD73 antibodies. The results of this study exhibited that CD73 blockade can neutralize the purinergic-mediated immunosuppression and enhance antitumor cytotoxicity of NKG2D-CAR-NK cells in both in vitro and in vivo conditions against solid tumor [137]. In another study, NK-cells derived from iPSCs were modified genetically using the piggyBac system to introduce them a CAR construct containing the transmembrane domain of NKG2D, the 2B4 co-stimulatory domain, and the CD3ζ signaling domain. The resulted NK-CAR-iPSC-NK cells could successfully mediate strong anti-tumor activity and repressed tumor growth and prolonged survival compared to PB-NK cells, iPSC-NK cells, or T-CAR-iPSC-NK cells (Fig. 2) [102].

### Pre-clinical and clinical studies

#### Pre-clinical studies in hematological malignancies

CAR-based immunotherapy in hematological malignancies such as leukemia and lymphoma has been probably the most efficient therapeutic method to date because of its impressive power to elicit an anti-tumor response. CAR-NK cells and CAR-T cells both have been successful to open new clinically relevant opportunities for the treatment of these malignancies.

**Fig. 3:**
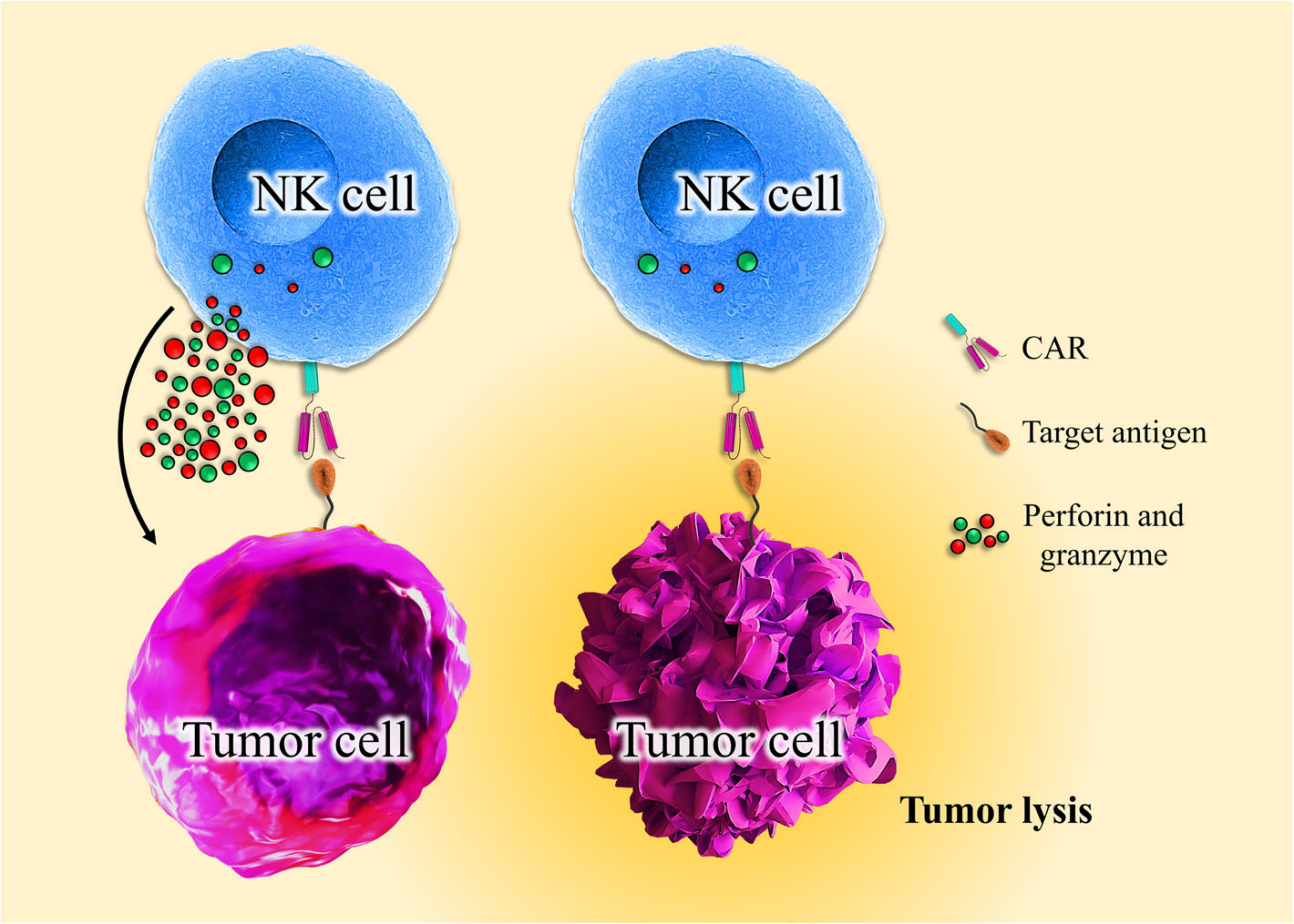
Engineered activatory receptors of NK cells strengthen NK cells in tumor killing. CAR redirect tumor cell-specific NK cells to bind to their specific target on the surface of the cancer cell, resulting in NK cell activation, cytolytic factors secretion, and tumor lysis

Many ex vivo studies have investigated the efficacy of CAR-NK cells, and some of them are still in progress to improve the efficacy of CAR constructs. Many of the earlier preclinical studies of CAR-NK cells focused on targeting B cell malignancies through utilizing the anti-CD19 and CD20-CARs [128, 129, 138]. In one study, CAR-NK cells were successfully produced by transduction of a CD19-specific CAR in hematopoietic stem/progenitor cells (HSPCs) and then differentiation into CAR-NK cells. The CAR-NK cells then could target the CD19-positive cells in B-linage malignancies [139] (Please see Fig. 3).

According to some evidence, acute and chronic B cell leukemias tend to develop resistance against normal/unmodified NK cells, especially after repeated and inappropriate treatment. To overcome the resistance, first-generation CD19-specific CAR NK cells were designed to target CD19-positive leukemic cell lines and primary leukemia cells [76]. It was also reported that CD19-specific CAR-expressing NK-92 cells cause efficient lysis of CD19-positive B-precursor leukemia cell lines and lymphoblasts in leukemia patients [76]. In another study, NK-92 cells were equipped with first-generation CAR targeting CD20 antigen which also contains CD3 ζ chain as intracellular signaling moiety. Similar to results observed for CD19-specific CAR-NK, this CD20-specific CAR NK-92 showed noticeably enhanced cytotoxicity against CD20-positive malignant cells including the cancer cells resistant to primary NK cells in primary lymphoma and leukemia cells while remained non-toxic to CD20-negative cells with expression of CD20 [140] (109).

In vivo studies using CD19-CAR-NK92 cells have shown the efficient distribution of the cells throughout the circulation and also good killing activity in leukemia murine models [75, 138]. In other recent studies, the mechanistic rationale of the NK-based therapy in bNHL (B cell non-Hodgkin’s lymphoma) was investigated. Also, the therapeutic potency and cell-cell interaction kinetics of CD19-CAR-NK cells as the “off the shelf” therapy were studied in vitro and in vivo models: bNHL cell lines, primary patient-derived cells, and in vivo xenograft model. Besides, systems biology transcriptomic analyses of flow-sorted lymphoma cells co-cultured with CD19-CAR-NK92 cells revealed activation of important pathways involved in lymphoma cells: IFNγ signaling, apoptosis mechanisms, ligand binding, and immunoregulatory and chemokine signaling pathways. Therefore, CD19-CAR-NK92 could trigger significant anti-lymphoma activity across a host of sensitive and resistant lymphoma cells that involved distinct NK cell-lymphoma cell interaction-mediated activation of cell death mechanisms [141] (Fig. 3).

Adoptive immunotherapy by (CAR)-engineered NK cells has been also examined for anti-cancer activity against Pre-B cell acute lymphoblastic leukemia (Pre-B-ALL). Exposure of Pre-B-ALL cell lines and primary blasts to FLT3-specific CAR NK-92 cells resulted in notable selective cytotoxicity of NK cells against them. Besides, FLT3-specific CAR NK-92 was effective in an SEM Pre-B-ALL xenograft model in NOD-SCID IL2R γ^null^ mice to exert high antileukemic activity and inhibit cancer progression [142].

Furthermore, the anti-cancer efficiency of CAR-NK has also been studied in non-Hodgkin’s lymphomas (NHLs) with poor prognosis such as aggressive T cell malignancies. It was found that CD5 or CD3 specific CAR-NK92 cells can maintain their stable expansion [98, 143] and exert efficient anti-tumor activity toward T cell leukemia, lymphoma cell lines, and primary malignant cells [98, 143].

MM as another type of malignant hematological cancer is characterized by abnormal proliferation of plasma cells in the bone borrow. These abnormal plasma cells then produce and secrete abnormal antibodies which can be detected in the serum or urine [144]. Application of CS1-specific CAR NK cells in MM was studied and tested for their potential anti-tumor activity in vitro and in vivo in MM-xenograft mouse models. The study used allogenic NK cells as a source for the development of CAR-NK cells. Using this type of cell may help to inhibit GvHD and increase NK cytotoxicity due to having non-matching killer immunoglobulin-like receptors (KIRs) [81, 145]. CS1-specific CAR-NK92 cells were generated by introducing an anti-CS1 CAR construct containing CD28-CD3 ζ as a costimulatory domain into NK cells. It has been shown that the production of IFN-γ was enhanced in vitro and tumor-killing activity in SCID gamma mice was induced [145]. CD138 is another antigen involved in MM development and proliferation and thus is a promising target for making CAR constructs. In a study, CD138-specific CAR NK-92MI displayed enhanced killing against CD138-positive human MM cell lines such as RPMI8226, U266, and NCI-H929 at various effector-to-target ratios [146]. Moreover, CAR-NK targeting CD138 has been successfully utilized in vivo experiments: xenograft NOD-SCID murine models [146].

#### Preclinical studies on solid tumors

CAR NK cell-based immunotherapies are also a promising therapeutic option for solid tumors. Glioblastoma (GB) is one of the most aggressive primary malignancies that can affect the central nervous system (CNS) [147]. Several GB-associated antigens have been incorporated in generating the CAR constructs. These most investigated GB-associated antigens include EGFR, EGFRvIII, and ERBB2 [130, 148, 149]. The therapeutic capacity of CAR-engineered NK cells for the treatment of GB has so far been studied by effector cells targeting EGFR, EGFRvIII, and ERBB2.

It has been shown that NK cell line YTS transduced with EGFRvIII-specific CAR construct can significantly display higher anti-cancer activity than the non-transduced cells which have no CAR. In this study, a CAR structure was designed and produced, consisting two parts, an antibody named MR1-1 with a scFv fragment capable of targeting EGFRvIII, and an intracellular part called DNAX-activating protein-12 (DAP12) to transfer the received signals into the cell [150]. DAP12 comprises an immunoreceptor tyrosine-based activation motif (ITAM) that transmits the activation signals for the NK cells [151]. Also, a study was shown that co-expression of the chemokine receptor CXCR4 in the EGFRvIII-specific CAR-NK cells improves the anti-cancer activity by enhancing tumor detection and homing properties of CAR-NK cells. These cells could trigger better inhibition of tumor growth and extended survival in EGFRvIII-positive GB xenografts in mice [150].

In another study, KHYG-1 NK cells with EGFRvIII-specific CAR showed good cytolytic activity against cancer cells [152]. In another study, a second-generation CAR containing EGFRvIII-specific antibody MR1-1 as cancer-targeting domain and CD28 and CD3ζ as signaling domains was used for transduction into the NK cell [148]. With the help of CARs, these engineered NK cells were redirected toward EGFRvIII-positive GB cells, resulted in quick and effective tumor cell killing. Moreover, EGFRvIII targeting NK-92 cells were frequently injected with the help of stereotactic method into orthotopic EGFRvIII-bearing GB xenografts in immunodeficient NSG mice which resulted in improved survival rate [148].

The receptor tyrosine kinase ErbB2 belongs to the same receptor family that EGFRvIII and EGFR are present, i.e., EGFR-related family of receptor tyrosine kinases. ErbB2 has also been incorporated in CAR-engineered NK-92 cells to be evaluated in cancer experiments [153]. It was found that ErbB2 is overexpressed in some types of human tumors with an epithelial origin and it is thought that it has a role in cancer development and progression [4, 154]. Several CAR-engineering approaches capable of targeting the ErbB2-positive cancers are now in development process. The suitability of CAR-based treatments for targeted cancer therapy was confirmed in both preclinical and clinical studies [155–157]. In this line, the genetically modified NK-92-scFv (FRP5)-zeta cells showed a great promise in killing the several types of ErbB2-expressing tumor cells and introduced as a new therapeutic solution for the treatment of ErbB2-expressing tumors [158].

In some preclinical studies, NK-92 cells were transduced with second-generation CARs containing a composite CD28-CD3ζ signaling domain to target ErbB2-positive cancer cells. These new CARs showed the strong and selective cytotoxicity against ErbB2-bearing target cells originated from different types of solid tumors such as the GB-related cell lines and primary GB stem cell cultures [153, 159]. Repeated stereotactic injection of these CAR-NK cells into the tumor location in orthotopic GB xenograft models efficiently repressed tumor progression and led to a marked prolongation of survival time [153]. In a study, the majority of mice whose had normal immune system and carrying syngeneic intracranial GL261/ErbB2 glioblastomas showed powerful endogenous antitumor immunity after intratumoral injection of NK-92/5.28. z cells. Unchanged NK-92 cells failed to inhibit tumor progression [153, 160]. Advanced renal cell carcinoma (RCC) is another solid tumor that has been evaluated with antigen-specific CAR-NK cells [161]. In this regard, a carbonic anhydrase IX (CAIX)-specific third-generation CAR-NK92 cells were utilized. In vitro consideration indicated that CAR-NK92 had a remarkable cytotoxic effect on target cells. Furthermore, a combination of bortezomib with CAR-NK92 cells showed greater suppressive efficacy against CAIX-positive tumor xenografts in comparison to the monotherapy with either CAR-NK92 cells or bortezomib [161].

Another group, Qing Zhang et al. constructed specific second-generation CAR-NK92 cells against an epithelial cell adhesion molecule- (EpCAM-) in colorectal cancer cells (CRC) [162]. In vitro results exhibited that EpCAM-specific CAR-NK-92 cells have a high potential cytotoxic effect against CRC cells. On the other hand, they reported a novel regimen, combination therapy with EpCAM-specific CARNK-92 cells and regorafenib, which increased anti-tumor efficacy to treat colorectal cancer in mouse models [162]. The synergy between the Robo1 BiCAR-NK immunotherapy and ^125^I has been examined in a murine model of orthotopic pancreatic tumor. The results illustrated that those groups treated with ^125^I seed +CAR-NK had higher tumor repression than ^125^I seed treatment alone. Consequently, this study indicated that ^125^I seed brachytherapy accompanied with Robo1-specific CAR-NK immunotherapy leads to better anti-cancer efficacy in a murine model of orthotopic pancreatic cancer [163]. A novel combination therapy consisting EGFR-CAR-NK-92 cells and a type of oncolytic virus was very effective against breast cancer brain metastases and could significantly increase survival time of tumor-bearing mice [164]. Reprogramming of NK cells has also been tested in proliferative prostate cells. CAR targeting PSCA which comprises DAP12 has been invented with NK cell line YTS [151]. Contacting of PSCA and anti-PSCA scFv (AM1) resulted in phosphorylation of DAP12-associated ZAP-70 kinase and enhanced IFN-γ production [151]. Besides, GD_2_-specific CAR NK cells in primary donor-drive NK cells and NK-92 cell line which carrying first- or second-generation CARs have been utilized in preclinical in vitro and in vivo models of neuroblastoma [165, 166] and Ewing sarcoma [167] which had effective antitumor activity. The introduction of CARs represents the most studied and developed approach that has recently reached preclinical evaluation (Table 1).

**Table 1:** Preclinical trials of CAR-engineered NK cells

Hematological malignancies					
Target	Malignancy	Signaling domain	Source of NK cells	Genetic modification method	Reference
CD19	B cell acute lymphoblastic leukemia (B-ALL)/B cell malignancies	CD28 + CD3ζ	Primary human NK cells from PBMCs	Lentiviral transduction	[168]
CD19	B cell acute lymphoblastic leukemia (B-ALL)/B cell malignancies	2B4 + CD3ζ	Primary human NK cells from PBMCs	retroviral transduction	[165]
CD19	B cell acute lymphoblastic leukemia (B-ALL)/B cell malignancies	4-1BB + CD3ζ	Primary human NK cells from PBMCs	mRNA electroporation	[128]
CD19	(B-ALL) and non-Hodgkin lymphoma (NHL)/B cell malignancies	4-1BB + CD3ζ	Primary human NK cells from PBMCs	mRNA electroporation; retroviral transduction	[129]
CD19	B-ALL cells/ B cell malignancies	4-1BB/CD3ζ	Primary human NK cells from PBMCs	Trogocytosis	[134]
CD19	B cell malignancies	CD3ζ	UCB	Retroviral transduction	[55]
CD19	B cell malignancies	CD3ζ	NK-92 cells	Retroviral transduction	[76]
CD19	CLL/ B cell malignancies	CD3ζ	NK-92 cells	mRNA electroporation	[75]
CD19	B cell malignancies	CD3ζ CD28 + CD3ζ 4-1BB + CD3ζ	NK-92 cells	Lentiviral transduction	[169]
CD19	CD20 resistant non-Hodgkin lymphoma (bNHL)	CD28 + CD3ζ	NK-92 cells	NR	[141]
CD20	B cell malignancies non–Hodgkin lymphoma (bNHL)	4-1BB + CD3ζ	Primary human NK cells from PBMCs	mRNA electroporation	[170]
CD20	B cell malignancies	CD3ζ	NK-92 cells	Retroviral transduction	[140]
CD19 and CD20	B cell malignancies	CD3ζ	NK-92 cells	Lentiviral transduction	[171]
CS1	Multiple myeloma	CD28 + CD3ζ	NK-92 cells	Lentiviral transduction	[145]
CD138	Multiple myeloma	CD3ζ	NK-92MI cells	Lentiviral transduction	[146]
FLT3	B-ALL/B cell malignancies	CD28 + CD3ζ	NK-92 cells	Lentiviral transduction	[142]
CD5	T cell malignancies	CD28 + 4-1BB + CD3ζ	NK-92 cells	Lentiviral transduction	[73]
CD5	T cell malignancies	CD28 + CD3ζ	NK-92 cells	Lentiviral transduction	[172]
CD3	T cell malignancies	CD28 + 4-1BB + CD3ζ	NK-92 cells	Lentiviral transduction	[98]
CD4	T cell malignancies	CD28 + 4-1BB + CD3ζ	NK-92 cells	Lentiviral transduction	[143]
EBNA3C	EBV-positive T cells	4-1BB + CD3ζ	NK-92MI cells	Retroviral transduction	[173]
Solid tumors					
GD2	Ewing sarcoma	CD28 + 4-1BB + CD3ζ	Primary human NK cells from PBMCs	Retroviral transduction	[167]
GD2	Neuroblastoma	CD3ζ	NK-92 cells	Retroviral transduction	[166]
GD2	Neuroblastoma	2B4 + CD3ζ	Primary NK cells from PBMC and NK-92 cells	Retroviral transduction	[165]
EpCAM	Breast cancer	CD28 + CD3ζ	NK-92 and NKL cells	Lentiviral transduction	[174]
EpCAM	Colorectal cancer	4-1BB + CD3ζ	NK-92 cells	Lentiviral transduction	[162]
Carbonic Anhydrase IX (CAIX)	Renal cell carcinoma (RCC)	CD28 + 4-1BB + CD3ζ	NK-92 cells	Lentiviral transduction	[175]
PSCA	Prostate cancer/PSCA^+^ tumor cells	DAP12	Primary human NK cells from PBMCs and YTS cells	Lentiviral transduction	[80]
GPA7	Melanoma	CD3ζ	NK-92 cells	mRNA electroporation	[176]
GPC3	Hepatocellular carcinoma (HCC)	CD28 + CD3ζ	NK-92 and Primary human NK cells from PBMCs	Lentiviral transduction	[177]
CEA	Colorectal cancer	CD3ζ	NK-92MI cells	Retroviral transduction	[178]
EGFR, EGFRvIII	Glioblastoma multiform (GBM)	CD28 + CD3ζ	NK-92 and NKL and Primary human NK cells from PBMCs	Lentiviral transduction	[130, 148]
EGFRvIII	Glioblastoma	DAP12	YTS cells	Lentiviral transduction	[78]
NKG2D	Osteosarcoma; prostate, colon, HCC, and breast cancer (NKG2D)	DAP10 + CD3ζ	Primary NK cells from PBMC	Retroviral transduction	[179]
NKG2D	CD73^+^ solid tumors /lung cancer	DAP10 + CD3ζ	NK-92 cells	PiggyBac transposon	[137]
NKG2D	Ovarian cancer	2B4 + CD3ζ	iPSC-derived human NK cells	PiggyBac transposon	[102]
ErbB2/HER-2	Breast/ovarian cancer/squamous cell carcinoma	CD3ζ	NK-92 cells	Retroviral transduction	[158]
ErbB2/HER-2	Ovarian and breast cancer	CD28 + CD3ζ	Primary NK cells from PBMC	Retroviral transduction	[180]
ErbB2/HER-2	Breast cancer/renal cell carcinoma (RCC)	CD28 + 4-1BB + CD3ζ	NK-92 cells	Lentiviral transduction	[159]
ErbB2/HER-2	Glioblastoma	CD28 + CD3ζ	NK-92 cells	Lentiviral transduction	[149]
ErbB2/HER-2	Breast cancer	CD28 + CD3ζ	NK-92 cells	mRNA electroporation	[181]
EGFR	Renal cell carcinoma (RCC)/solid tumor	CD28 + 4-1BB + CD3ζ	NK-92 cells	Lentiviral transduction	[182]
EGFR	Breast cancer brain metastases	CD28 + CD3ζ	Primary NK cells from PBMC and NK-92 cells	Lentiviral transduction	[164]

*EGFR* epidermal growth factor receptor, *HCC* hepatocellular carcinoma, *PBMC* peripheral blood mononuclear cell, *CEA* carcinoembryonic antigen, *RCC* renal cell carcinoma, *CAIX* carbonic anhydrase IX, *UCB* umbilical cord blood

### Clinical study

Although CAR T cell therapy has shown promising results in the treatment of acute lymphoblastic leukemia the method has not been so good at the treatment of acute myeloid leukemia. As an alternative approach, CD33-directed CAR-NK-92 cells were evaluated in phase 1 clinical trial in patients with refractory acute myeloid leukemia without major adverse effects, indicating that CAR-NK cells may be a safe alternative for CAR-T cells [183]. However, CAR-NK cells have some limitations such as short life-time, low capacity to infiltrate into the tumor sites, and weak cytotoxicity in vivo [184]. To improve the proliferation capacity and persistence of NK cells, an optimized CAR-NK cell construct named CD19-CD28-zeta-2A-iCasp9-IL15 comprised the coding sequence of IL-15 was transduced into cord blood natural killer (CB-NK) cells. The optimized CAR-NK cells showed great potency in destroying the cancer cells in patients with relapsed/refractory CD19+ B lymphoid malignancies (NCT03056339).

Furthermore, to evaluate the safety and feasibility of CAR-NK cell treatment in patients with metastatic solid tumors phase 1 of a clinical trial are now in progress. For this aim, CAR-NK cells targeting NKG2D ligands are being utilized and following infusion of CAR-NK cells interleukin-2 (IL-2) will be injected subcutaneously into some patients to support the survival of CAR-NK cells in vivo (NCT03415100).

Treatment with Robo1 BiCAR-NK could improve ^125^I seed brachytherapy in vitro and in vivo in pancreatic cancer models [163]. It has been shown that this combination therapy resulted in higher tumor reduction significantly. For this purpose, Robo1 BiCAR-NK92-mediated immunotherapy is now tested in phase I/II clinical trials in patients with relapsed and refractory pancreatic cancer (NCT03941457). Details have been shown in Table 2.

**Table 2:** Robo1 BiCAR- NK92-mediated immunotherapy tested in phase I/II clinical trials in patients with relapsed and refractory pancreatic cancer

Clinical trial identifier	Target antigen	Condition or disease	Origin of NK cell	Construct	Phase	Status	Location
NCT03824964	CD19/CD22	Refractory B cell lymphoma	Allogeneic NK cells	Not mentioned	Early phase I	Not yet recruiting	Not mentioned
NCT03940833	BCMA	Multiple myeloma	NK 92 cells	Not mentioned	I/II	Recruiting	Suzhou, Jiangsu, China
NCT02892695	CD19	Lymphoma and leukemia	NK 92 cells	Not mentioned	I/II	Unknown	Suzhou, Jiangsu, China
NCT03579927	CD19	Lymphoma and leukemia	Cord Blood NK Cells	CAR.CD19-CD28-zeta-2A-iCasp9-IL15	I/II	Not yet recruiting	Houston, TX, USA
NCT03692663	PSMA	Castration-resistant Prostate Cancer	Not specified	Not mentioned	Early phase I	Not yet recruiting	Not mentioned
NCT03692637	Mesothelin	Epithelial ovarian cancer	Not specified	Not mentioned	Early phase I	Not yet recruiting	Not mentioned
NCT03941457	ROBO1	Pancreatic cancer	Not specified	BiCAR-NK cells	I/II	Recruiting	Shanghai, China
NCT03940820	ROBO1	Solid tumor	Not specified	Not mentioned	I/II	Recruiting	Suzhou, Jiangsu, China
NCT03415100	NKG2D ligands	Solid tumors	Autologous or allogeneic NK cells	Not mentioned	I	Recruiting	Guangzhou, Guangdong, China
NCT03056339	CD19	Lymphoma and leukemia (relapsed/refractory B cell malignancy)	Umbilical cord blood I	CAR.CD19-CD28-zeta-2A-iCasp9-IL15	I/II	Recruiting	Houston, TX, USA
NCT02944162	CD33	Acute myeloid leukemia (AML)	NK 92 cells	CAR.CD28, CD137 and CD3 zeta	I/II	Unknown	Suzhou, Jiangsu, China
NCT03931720	ROBO1	Malignant tumor	Not specified	BiCAR-NK/T cells	I/II	Recruiting	Suzhou, Jiangsu, China
NCT03692767	CD22	Refractory B cell lymphoma	Allogeneic NK cells	Not mentioned	Early phase I	Not yet recruiting	Not mentioned
NCT03690310	CD19	Refractory B cell lymphoma	Allogeneic NK cells	Not mentioned	Early phase I	Not yet recruiting	Not mentioned
NCT01974479	CD19	All	Haploidentical donor NK cells	Not mentioned	I	Suspended	Singapore
NCT00995137	CD19	All	Expanded donor NK cells	Not mentioned	I	Completed	Memphis, TN, USA

### Challenges of using CAR-NK cells and strategies for improving their functions

#### Efficacy of transduction methods

Despite the many advantages of using NK cells, some challenges limit these effector cells to generate CAR-modified NK cells for clinical application [185]. To begin with, the efficacy of transduction methods is one of the challenges to be considered since lentiviral vectors (LVs) have just received some attention as a vehicle for CAR transduction with high potential and high efficacy in clinical uses [53, 186, 187]. However, these vectors have the risk of inducing mutagenesis, so is not desirable for human [188]. On the other hand, in non-viral methods such as mRNA electroporation, the transcript cannot integrate into the genome because of its short-expression time [128]. Moreover, larger quantities of mRNA would be required for clinical applications that multiple infusions would be necessary to make up for the transient expression of the CAR proteins. As stated by some studies, it has been shown that transduction method efficiency percentages are variable in various sources of NK cells [119]. In this regard, one study reported that the efficiency of primary CB or PB-derived NK cells that were transfected by mRNA was < 10% [119, 189]. Viral transduction methods are very weak at transducing CAR into freshly isolated PB NK cells. However, it seems that NK cells at their activation and expansion stage are more susceptible to viral transduction methods. Much better transduction rate was achieved when NK cells from PB (12–73%) [119] or in one study CB were activated and expanded (median 69%; range 43–93%) [79] and 80% (range 67–96%) in another study [179].

Another concern about immunotherapy with NK cells goes back to using allogeneic NK cells, which has the possibility of infusion contamination with T or B cells in the expanded NK cell preparation, which can initiate undesirable immune responses in receiver’s body such as the GVHD or post-transplant lymphoproliferative disease [81].

#### Potential challenges of NK cell expansion

Furthermore, one more potential challenge to use NK cell-based immunotherapy is keeping NK cell expansion in the host’s body after infusion. Because these cells are short-lived, it is necessary to use a new strategy for maintaining their numbers and activity [106, 190]. Recently, the administration of exogenous cytokines has been opening new prospects to tackle this issue. Regular IL-2 injection is an example to maintain function and biology of NK cells as desire; however, it can also induce regulatory T cell proliferation which suppresses NK cell functions [190, 191]. Therefore, other cytokines that can selectively activate NK cells have been explored [192]. On the report of a study, patients who were treated with strong lymphodepletion chemotherapy before NK cell infusion by using high-dose cyclophosphamide and fludarabine took advantage of better anti-cancer activity of NK cells and successful expansion of infused NK cells. In patients receiving high-dose lymphodepletion chemotherapy, a high level of endogenous IL-15 was observed indicating an important role of IL-15 in NK-cells expansion [89]. It was known that IL-15 does not induce Treg expansion [193]. Although exogenous IL-15 can lead to toxicities such as grade 3 hypotension, thrombocytopenia, and elevations of Alanine Transaminase (ALT) and Aspartate Transaminase (AST) and neutropenia, but these side-effects are dose-dependent. Therefore, IL-15 as an activator of NK cells can be safely administered to patients with malignant and poor-prognosis/metastatic cancers by using alternative dosing strategies such as continuous intravenous infusions and subcutaneous administration [194]. IL-12 is another pro-inflammatory cytokine with known immunomodulatory properties on NK cells and lymphoid cells. In preclinical models, administration of IL-12 augmented migratory capacity and biologic functions of NK cells [195]. However, there are still serious toxicity concerns about the clinical application of IL-12 due to its pleiotropic nature [196].

Another new approach is the incorporation of both IL-2 and IL-15 genes into the CAR constructs to be expressed and secreted within the TME. This approach has been conducted in a mouse model of Raji lymphoma. The results showed that a single infusion of CAR.19/IL15+ CB-NK cells can control tumor progression and increase survival through improving their cancer recognition and cytotoxic activity in comparison to CAR.19/IL15-negative NK cells. These results indicate the pivotal impact of IL-15 on the antitumor activity of NK cells by improving their expansion and persistence capacity [55].

#### Efficacy and safety

The CAR-engineered immune effector cells (CAR-T and CAR-NK) are developed over the last two decades as the adoptive cell therapy of cancers. However, their therapeutic efficacy against solid tumors remained restricted. Consequently, some new solutions and approaches are being evaluated to improve the effectiveness of these cells.

One of the recently developed methods for enhancing the efficacy of CAR-NK cells in a solid tumor is utilizing chemotherapy drugs in combination with CAR NK cells. Qing Zhang and his colleagues for the first time have determined the synergistic effects of Cabozantinib and EGFR-specific CAR-NK-92 cells in vitro and in vivo. Cabozantinib is a multikinase inhibitor that has repressing effects on myeloid-derived suppressor cells and Tregs and also is synergistic in combination with immunotherapies in the treatment of cancers [197, 198]. Therefore, a combination of Cabozantinib with CAR-NK-92 cells can be used to overcome the immunosuppressive environment of tumors. It was shown that the efficacy of the CAR-NK-92 cells increases in combination therapy with Cabozantinib against human renal cell carcinoma xenograft models [182]. On the other hand, CAR-engineered T cell therapy in clinical cases caused severe side effects [199, 200], whereas no such serious side effects were reported about CAR-NK cell therapy. For this reason, various studies have been conducted to further increase the effectiveness and safety of CAR-NK cell therapies and combination therapies [33, 164, 201].

Generally, mature NK cells have limited persistence in the tumor site, so permanent side effects are not observed. Nonetheless, there are types of NK cells with higher long-lived, such as those derived from cord blood or hematopoietic stem cells as that may cause longstanding toxicity [202]. To address this issue, today, a new method that incorporating caspase-controlled suicide vectors into CAR-NK cells has been studying, which could rapidly eradicate the transduced cells. In these regards, a recent study illustrated that applying the inducible caspase 9 (iCAS-9) suicide systems in CD19-CAR+IL15 NK cells construct causing apoptosis within 4 h upon addition of the matching small molecule dimerizer [55].

## Conclusion and perspective

CAR-engineered NK cells can be successfully employed to improve the therapeutic outcome of cell-based cancer immunotherapy. They have been investigated in many research and also clinical trials with acceptable therapeutic results while keeping the side-effect at the minimum level in comparison to other cell-based immunotherapies. However, technical and biological challenges are still associated with gene delivery into NK cells and therefore have hindered this approach to become a routine anti-cancer treatment at the moment. There are still some difficulties in CAR delivery into NK cells and in NK cells’ persistence and expansion within the body after infusion. Despite all these problems, CAR NK cells have gained much attention due to their lower safety concerns and the possibility of triggering negative immune reactions such as GvHD. NK cells can provide a homogenous, off-the-shelf, standardized cell-based medicine for the treatment of patients with resistant/hardly treatable cancers. CAR NK cells have come to light as top anti-cancer agents with great effectiveness; however, for attaining this goal, improved CRA-NK cells with fewer side effects are required. Besides, we need better transfection methods, better than routine NK cell sources that are much more safer and efficient for CAR NK cells. Many advanced strategies including further optimization of transfer methods, novel CAR construction and delivery methods such as novel gene-editing techniques based on CRISPR/Cas9, and finally combination of CAR NK cells with other treatments such as the checkpoint inhibitors, mAbs, drugs targeting TME, and chemotherapies. These strategies may strengthen the immune system overcome cancer and open a new chapter in cancer treatment. With the improvement of CAR NK cells, it is not surprising that in the near future, CAR-NK cell-based immunotherapy will revolutionize the treatment of cancer.

## Data Availability

Not applicable

## Abbreviations

NK: Natural killer
CAR: Chimeric antigen receptor
CAR-T: CAR-engineered T
CAR-NK: CAR-engineered NK
GVHD: Graft-versus-host disease
CRS: Cytokine release syndrome
TCR: T cell receptor
NCRs: Natural cytotoxicity receptors
ITAM: Immunoreceptor tyrosine-based activation motif
KIRs: Killer cell Ig-like receptors
ADCC: Antibody-dependent cell-mediated cytotoxicity
AML: Acute myeloid leukemia
scFv: Single-chain variable fragment
TME: Tumor microenvironment
iPSCs: Induced pluripotent stem cells
PB NK: VEGF, vascular endothelial growth factor
Tregs: Regulatory T cells
